# Combined Description of the Equation of State and Diffusion Coefficient of Liquid Water Using a Two-State Sanchez–Lacombe Approach

**DOI:** 10.3390/molecules28062560

**Published:** 2023-03-11

**Authors:** Valeriy V. Ginzburg, Enza Fazio, Carmelo Corsaro

**Affiliations:** 1 Department of Chemical Engineering and Materials Science, Michigan State University, East Lansing, MI 48824, USA; 2 Department of Mathematical and Computational Science, Physics Science and Earth Science, University of Messina, 98166 Messina, Italy

**Keywords:** water, diffusion, density, relaxation, fragile-to-strong transition, scaling, two-state theory

## Abstract

Water is one of the most important compounds on Earth, yet its material properties are still poorly understood. Here, we use a recently developed two-state, two-(time)scale (TS2) dynamic mean-field model combined with the two-state Sanchez–Lacombe (SL) thermodynamic theory in order to describe the equation of state (density as a function of temperature and pressure) and diffusivity of liquid water. In particular, it is shown that in a relatively wide temperature and pressure range (160 K < *T* < 360 K; 0 < *P* < 100 MPa), density and self-diffusion obey a special type of dynamic scaling, similar to the “τTV” scaling of Casalini and Roland, but with the negative exponent *γ*. The model predictions are consistent with experimental data. The new equation of state can be used for various process models and generalized to include multicomponent mixtures.

## 1. Introduction

Water is one of the most anomalous substances on Earth [1,2] as well as one of the most important in various industries—from chemical, to personal care, to pharmaceutical and medical; it is sometimes called the solvent of life [3,4]. Hydrophilicity and hydrophobicity are often critical design attributes for many novel materials [5,6]. Therefore, a better understanding of water’s properties at the molecular level is essential. However, to gain this understanding, one needs to consider multiple variables related to dynamics, thermodynamics, quantum mechanics, chemistry, and geometry [7,8]. For example, the formation of locally ordered structures in water, such as tetrahedral ones, is ascribed to the high directionality of the hydrogen bonds (HBs), whose strength and lifetime increase upon lowering the temperature below about 320 K [9]. On the other hand, the van der Waals interactions favor a denser and, therefore, more disordered configuration—especially at the highest temperatures [10]. Over the years, multiple theoretical and computational models of water have been proposed, although many disagreements still remain [1,2,7,11,12,13,14,15,16,17,18,19,20,21,22,23,24,25].

The most interesting property (or anomaly) of liquid water is that its density (at atmospheric pressure) exhibits a strongly non-monotonic temperature dependence (with a maximum at *T* ≈ 277 K), often understood in terms of competition between two or more competing local environments with different density and symmetry whose relative concentration is indeed determined by thermodynamic conditions [11,12,14,26]. Among many other water anomalies [1,27], we would like to recall that at atmospheric pressure, (1) the isobaric specific heat has a minimum at about *T* ≈ 308 K, (2) the isothermal compressibility has a minimum at about *T* ≈ 319 K, and (3) the coefficient of thermal expansion is negative below *T* ≈ 277 K. These anomalies can be understood, at least qualitatively, on the basis of two coexisting local structures of water and the associated idea of a liquid-liquid critical point (LLCP) [2,17,28,29,30]. However, there are still many unclear aspects regarding the liquid-liquid phase transition scenario, including the potential existence of a second critical point, as well as additional water anomalies [1,28,31,32,33,34].

The above discussion concentrated on thermodynamic properties; however, the dynamic and transport properties of supercooled liquid water are also extremely non-trivial. The dielectric α-relaxation time, viscosity, and self-diffusion coefficient all exhibit Arrhenius temperature dependence (at atmospheric pressure) for high temperatures (*T* > 280 K). At temperatures around *T* = 220 K, an abrupt so-called fragile-to-strong (FS) transition is observed [2,16,20,35,36,37,38,39,40,41,42]. The nature of this transition is still debated (see, e.g., a recent comprehensive computational study by Zhang et al. [43] of the Al_90_Sm_10_ metal glass); recently, it has been successfully described using a two-state framework [20,42]. Even though in a real material a potential energy landscape is a rugged hypersurface with multiple minima, one can simplify the analysis by only considering the two lowest energy minima and neglecting the contributions of the higher ones. For water in particular, Shi, Russo, and Tanaka [7,17,20] developed a simple combined two-state model for both density and transport properties, capturing many important details; however, many questions remained.

Recently, one of us proposed a two-state approach to describe the relaxation time and density of polymeric and low-molecular-weight glass-forming materials near their glass transition temperature [44,45,46,47,48]. Within this framework (labeled SL-TS2 approach), it is hypothesized that a glass-former is characterized by competition between two states with different activation energies, different mass, and different cohesive energy densities. At high temperatures, the material has Arrhenius-type dynamics, while at lower temperatures the relaxation time increases more dramatically upon cooling, due to the increasing fraction of the more sluggish, low-temperature state; this transition is clearly reminiscent of the hypothesized FS crossover in water. There are many other features of the new model that are quite similar to well-known two-state theories of liquid water. Thus, it is appealing to see how the SL-TS2 approach can be applied to describe liquid water, as well as what (if any) modifications need to be made to capture its anomalies.

One interesting feature of the SL-TS2 approach is its ability to explain dynamic scaling (see, e.g., Casalini, Roland, and co-workers [49,50,51,52,53,54,55]). This Casalini–Roland or “τTV” scaling suggests that for many materials, the *α*-relaxation time (*τ*_*α*_) depends not on the temperature (*T*) and pressure (*P*) separately, but on some combination thereof, i.e., *τ_α_* = *f*(*Tv_sp_^γ^*), where *v_sp_* is the specific volume (inverse of the density) at the current *T* and *P*, *f(x)* is some (material-dependent) function, and *γ* is a material-dependent constant whose value can be related to the effective interparticle interaction potentials [56]. Within SL-TS2, the relaxation time is assumed to be a function of the “state variable” *Z* = *T/T_X_(P)*, while the specific volume has the form *v_sp_* = *v*_0_*(P)g(Z)*. It is further assumed (in the limit of low pressures) that

$$T_{X}\left(P\right)=T_{X}\left(0\right)\exp\left[P/P_{0,T}\right]\;\mathrm{and}\;v_{0}\left(P\right)=v_{0}\left(0\right)\exp\left[P/P_{0,v}\right]$$

where the scaling exponent $\gamma =P_{0,v}/P_{0,T}$. For water, the original Casalini–Roland scaling (with positive *γ*) fails to describe the relaxation and density data. Is it possible, though, that some modified version of the same scaling could work even in this case?

**Figure 1:**
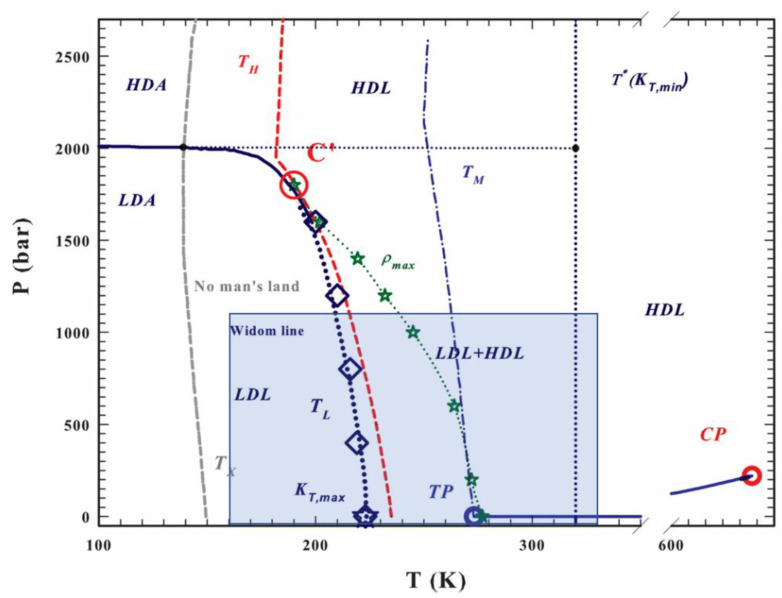
The qualitative phase diagram for amorphous water. Here, LDL is low-density liquid water, HDL is high-density liquid water, TP is the triple point, and CP is the critical point; for other abbreviations, see Ref. [57]. The blue rectangle highlights the region considered in this modeling study. Note that on the pressure scale, 1 bar ≈ 0.1 MPa (adapted from Ref. [57] with permission from AIP Publishing).

In this paper, we attempt to apply the SL-TS2 approach (with some modifications) to amorphous water; see Figure 1 for its qualitative phase diagram (adapted from Mallamace et al. [57]). The dynamic scaling, if any, is likely valid far away from the hypothesized LLCP, denoted as C’ in Figure 1 (*T_LLCP_*~200–220 K, *P_LLCP_*~180–200 MPa) [58]. We therefore concentrate on the low-pressure region, *P* < 100 MPa or 1000 bar, but consider a sufficiently broad temperature region—between 160 K and 360 K—to capture both the density anomalies and the fragile-to-strong transition. In Figure 1, this region is shown as a blue rectangle.

Below, we describe the Sanchez-Lacombe two-state, two-(time)scale (SL-TS2) model, the dynamic scaling, and the experimental data on the density and diffusion coefficient of liquid water. Our approach resembles that of Shi, Russo, and Tanaka [7] but relies on a different free energy expression and, thus, a different equation of state. The model predictions are compared with experimental results. Finally, we discuss the limitations of this approach, its potential modifications, and future work. 

## 2. Results 

### 2.1. Master Curves and Scaling

In Figure 1 (adapted from Mallamace et al. [57]), the qualitative phase diagram of amorphous water is shown, with various states and phase transition lines. As mentioned above, in our analysis we concentrate on the region shaded in blue (temperatures between 160 and 360 K; pressures between 0 and 1000 bar (~100 MPa)).

**Figure 2:**
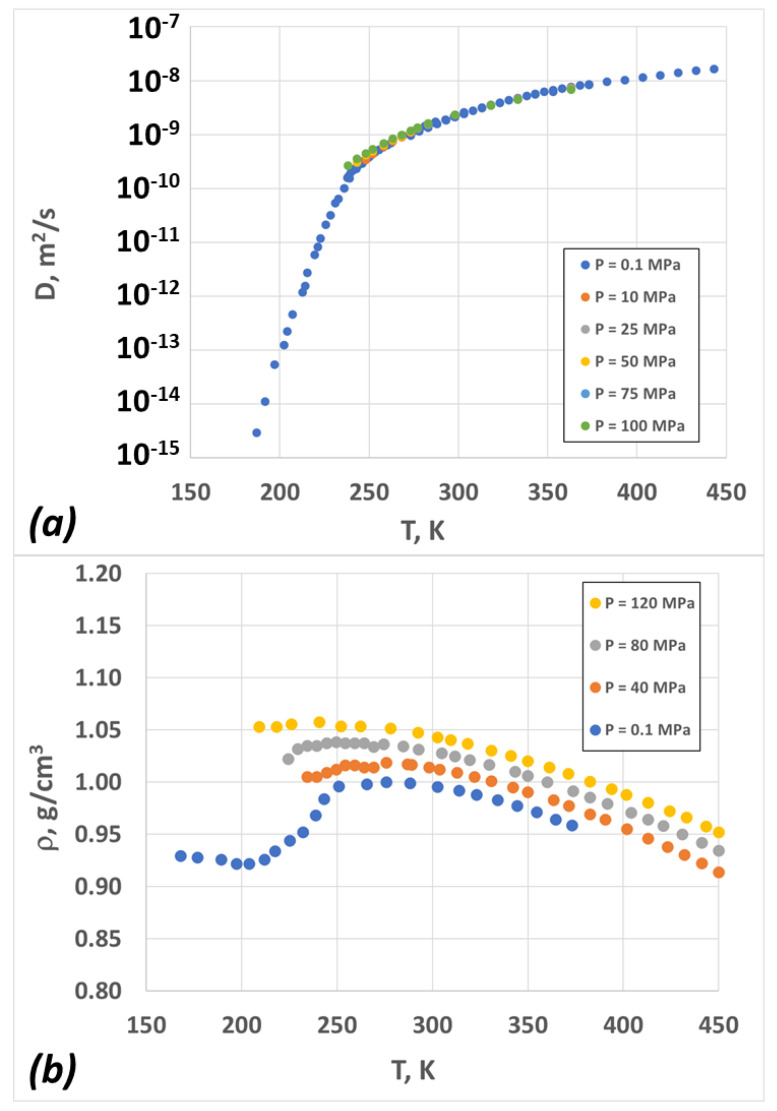
Experimental data for liquid water as a function of temperature at different pressures (see text for more details): (**a**) diffusion coefficient; (**b**) density.

The diffusion and density data for amorphous water are depicted in Figure 2 (for details about the experiments, the reader can refer to Section 4.4). The diffusion coefficient is nearly independent of pressure, while the density exhibits strong pressure dependence. Within the experimentally relevant temperature range, the density exhibits a minimum and a maximum for the atmospheric pressure case, but it almost monotonically decreases for the case of very high pressures (*P* > 100 MPa).

We applied the scaling procedure discussed above in order to create “master curves” for the scaled density and scaled diffusion coefficient as functions of scaled temperature. To do so, we first transformed the temperature, density, and diffusion coefficients to their “effective” values according to the following rules:

(1) $$T_{eff}=T\exp\left(a_{T}P\right)$$

(2) $$\rho_{eff}=\rho \exp\left(-a_{\rho }P\right)$$

(3) $$D_{eff}=D\exp\left(a_{D}P\right)$$

Note that the transformation of Equations (1)–(3) is consistent with the “τTV” dynamic scaling, at least in the narrow range of low (0–100 MPa) pressures. The scaling parameter *γ* is given by $\gamma =-a_{T}/a_{\rho }\approx -1.7\pm 0.25$. The negative sign of *γ* is due to the different pressure dependence of the density scale (positive) and the energy scale (negative). We discuss this interesting effect in more detail in the Section 3.

**Figure 3:**
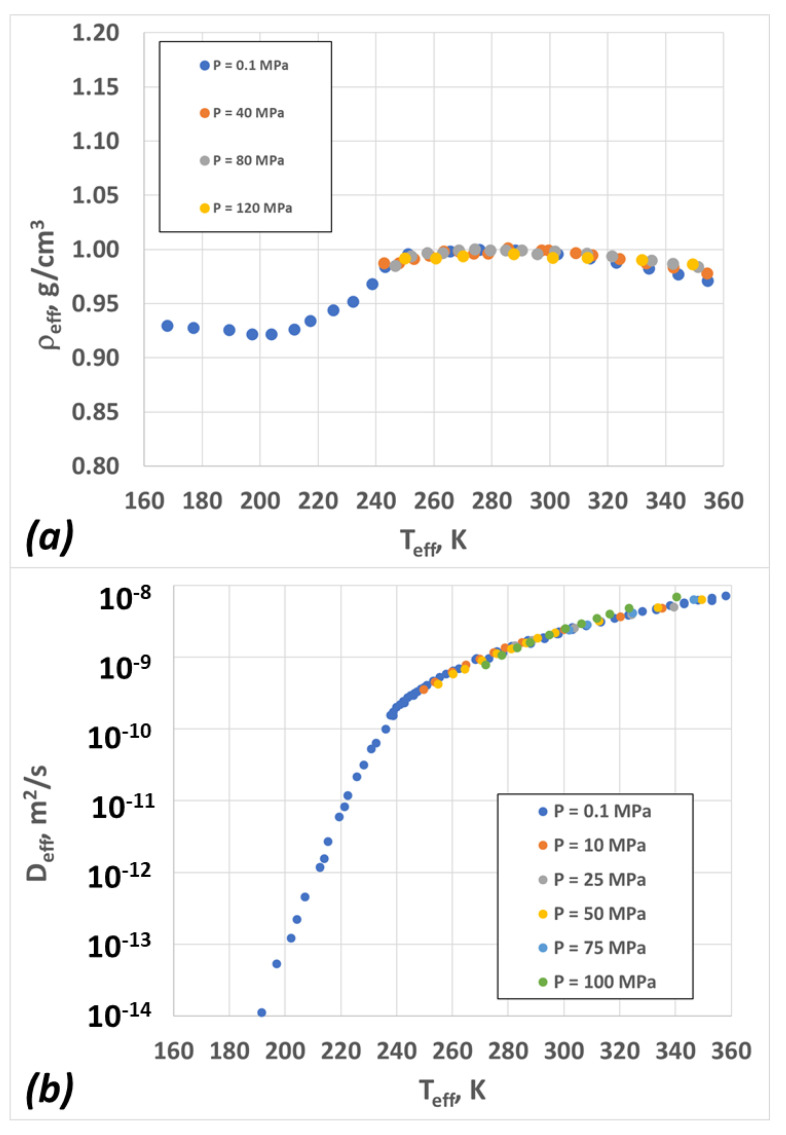
The data from Figure 2 rescaled according to Equations (1)–(3) (see text for more details): (**a**) density; (**b**) diffusion coefficient.

The data then collapse into “master curves”, as shown in Figure 3. The scaling parameters are summarized in Table 1. The collapse of the density curves is easily understood if we assume that the transition temperature *T_X_(P)* decreases with pressure according to Equation (16) (see below, in the Section 4). This contrasts with many other amorphous materials, where the glass transition temperature (*T_g_*) increases with *P*; there, the transition temperature (*T_X_*) and the glass transition temperature (*T_g_*) track one another quite closely. For water, this is not the case, and the exact location of its *T_g_* is still debated, although it should be in the order of 160 K or even less. [59] Thus, the density data for amorphous water at *T* > 160 K can be considered “equilibrium” (if we disregard the effects of crystallization) and modeled using an equilibrium theory. This analysis is discussed below.

### 2.2. Density vs. Temperature and Pressure—Model and Experiment

Using the SL-TS2 model, we can compute the equilibrium density vs. temperature and regress the parameters to obtain the best fit to the experimental data. The optimization was carried out using the Monte Carlo process, with initial guesses determined by trial and error. To reduce the number of independent parameters, we set *α_HH_* = *α_LH_* = 1. The best fit parameters are summarized in Table 2. The error in the parameter determination was about 5%.

**Figure 4:**
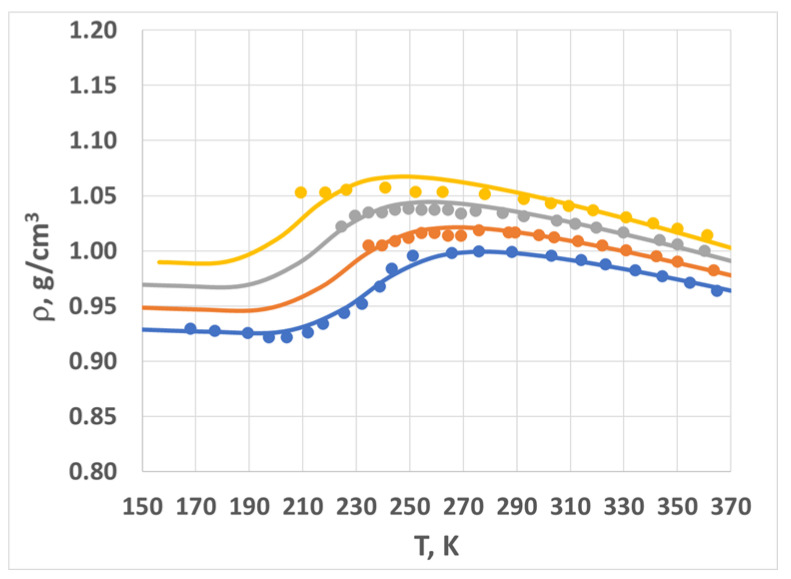
Density vs. temperature for multiple pressures: 0.1 MPa (blue), 40 MPa (orange), 80 MPa (grey), and 120 MPa (gold). Circles represent experimental data; lines are model predictions. See text for more details.

In Figure 4, we plot the density vs. temperature data and the model fit for multiple pressures. The ambient pressure data (blue circles) are plotted against the model’s best fit (blue line). The model correctly captured the initial density decrease (160–210 K), followed by the sharp increase (210–275 K) and then another decrease (for temperatures greater than 275 K). We then applied the scaling rules to calculate the density–temperature curves for several other pressures, represented by the orange (*P* = 40 MPa), grey (*P* = 80 MPa), and gold (*P* = 120 MPa) circles and lines.

The combination of the two-state Sanchez–Lacombe theory (see below in the Section 4) and the scaling rules (Equations (1)–(3)) provides an implicit equation of state for amorphous water in the specified range of temperatures and pressures (160 K < *T* < 360 K; 0 < *P* < 100 MPa). The agreement between the model and the measured data is fairly good—generally within experimental error (~1%). The model breaks down at high pressures and low temperatures; we address this further in the Section 3.

### 2.3. Diffusion Coefficient vs. Temperature and Pressure—Model and Experiment

**Figure 5:**
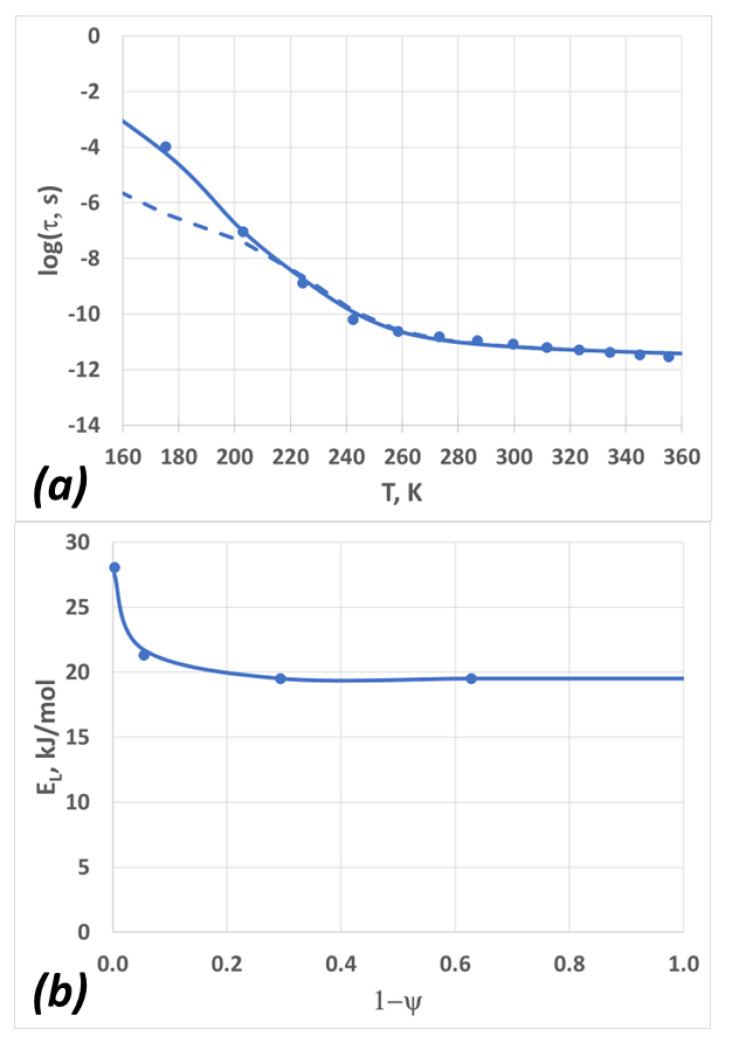
(**a**) The dependence of the relaxation time on temperature; circles are experimental data (converted from the diffusion coefficient data using Equation (8)), the dashed line is the TS2 fit, and the solid line is the “modified TS2” fit. (**b**) The dependence of the L-state activation energy on the H-state molar fraction, 1−*ψ*.

We began by considering the ambient pressure data and applying Equations (13) and (14) (see below, in the Section 4), setting *a*_0_ = 1.3 Å, so that *τ_α_*(*T* = 273 K) = 17 ps, consistent with the dielectric relaxation measurements of Bertolini et al. [60] and Buchner et al. [61] The model was then fitted to the experimental data using the generalized reduced gradient (GRG) nonlinear optimizer in Excel, and the best values for the TS2 parameters log(*τ_∞_*), *E_L_*, and *E_H_* were obtained. The error bars for log(*τ_∞_*) were estimated to be ±0.2, and the errors for *E_L_* and *E_H_* were about 15%. The best TS2 fit for the atmospheric pressure diffusion coefficient is given in Figure 5a (dashed line). The agreement between the model and the experiment results was good at temperatures between *T* = 200 K and *T* = 360 K, but it worsened significantly at lower temperatures. To specifically address the low-temperature behavior, one can stipulate that there is an additional dependence of *E_L_* on *ψ*, as follows:

(4) $$E_{L}\left(\psi \right)=E_{L,\min}+\left(E_{L,\max}-E_{L,\min}\right)\exp\left(-k\left[1-\psi \right]\right)$$

The results from this “modified TS2” are shown in Figure 5a (solid line), and the dependence of *E_L_* on *ψ* is depicted in Figure 5b. The model parameters are summarized in Table 3. Note that the “modified TS2” is consistent with the dynamic scaling, given that the relaxation time depends only on *ψ*, albeit this dependence is no longer linear. The functional form of Equation (2) is empirical; one could also use other forms, e.g., a two-state sigmoidal function. This would suggest a separate low-temperature transition caused by a possible rearrangement of hydrogen bonds within the L-clusters. At this time, there are not enough data to explore this topic further. 

**Figure 6:**
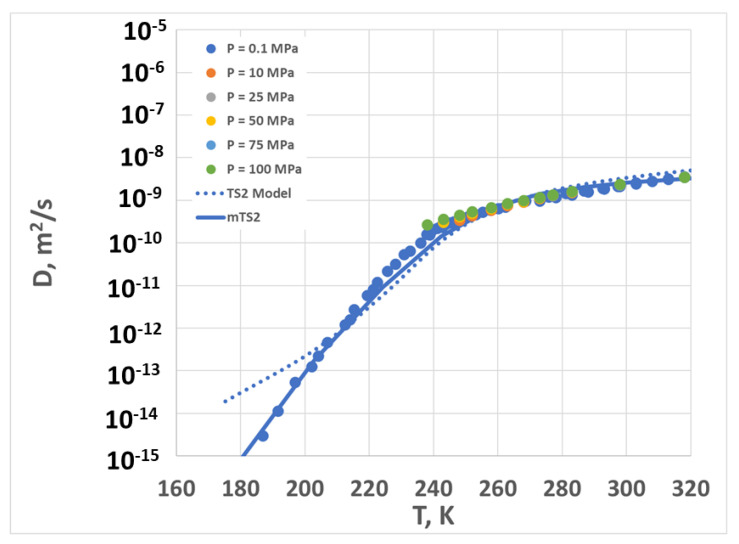
Diffusion coefficient vs. temperature for different pressures. Symbols are experimental data (corresponding to multiple pressures, as shown in the legend), the dotted line is the TS2 fit, and the solid line is the mTS2 fit.

Finally, we transformed the above results back to the calculation of the diffusion coefficient by using Equations (1)–(3) and (14) (see below, in the Section 4). The results are shown in Figure 6. Note that the data for different pressures nearly all collapse on top of one another, so a single model curve is shown for TS2 and another is shown for mTS2. Because of the scaling, the model curves for other pressures would collapse exactly on top of the atmospheric pressure line. The agreement between the model and the experimental results is fairly good (the average error in log(*D*) is less than 0.4, except for the TS2 model at temperatures below 200 K).

## 3. Discussion

We developed a new two-state model to describe the diffusion coefficient and the density of liquid (including supercooled liquid) water in a broad range of temperatures and pressures. The proposed two-state model is based on the Sanchez–Lacombe (SL) lattice approach developed previously for the analysis of polymeric and low-molecular-weight glass-formers. Unlike the earlier models, however, in the case of water, the low-temperature state of matter has a lower density (higher specific volume) than the high-temperature one. Within the SL framework, this means that the configurational entropy of the H-state is lower than that of the L-state, contrary to thermodynamics. Thus, we postulated that the overall entropy, in addition to the SL configurational entropy, contains the contribution from the hydrogen-bonding network, and that the hydrogen-bonding entropy of the H-state is significantly higher than that of the L-state, $S_{H,H}=S_{H,L}+\Delta S_{H}$. The model successfully captures the non-monotonic behavior of the density as a function of temperature. When combined with the TS2 relaxation time model, it is also able to capture the dependence of the diffusion coefficient on temperature—at least for *T* > 200 K. For temperatures in the range 160 K < *T* < 200 K, it is necessary to assume an additional change in the low-temperature activation energy (the “mTS2” model); the origins of this change are not yet known but could be due to some further changes in the nature of the hydrogen-bonding network. In fact, below 220 K, only the “mTS2” model is able to reproduce the experimental values of self-diffusion, and this may correspond to the crossing of the Widom line—the critical isochore emanating from the hypothesized LLCP of water towards lower pressures and higher temperatures (see Figure 1) [62].

We also demonstrated that water exhibits dynamic scaling, similar to the “τTV” scaling described by Casalini, Roland, and other authors for multiple glass-forming materials. One interesting distinction for water is as follows: The transition temperature from the low-T state to the high-T state (*T_X_*) is a decreasing function of pressure, while in most conventional glass-formers the glass transition temperature (*T_g_*) and the melting temperature (*T_m_*) are increasing functions of pressure. This anomaly is connected to the fact that the L-state has a lower density and the H-state has a higher density; thus, increasing the pressure makes the H-state more favorable (at constant temperature) and causes the L-H transition to shift towards lower temperatures. The scaling exponent *g* is estimated to be ~(−1.7), while for most other materials obeying the “τTV” scaling, *g* is usually positive. There are several theories relating *g* to the effective interaction between the molecules or clusters; however, those theories apply for positive values of *g.* A consistent molecular-theory-based understanding of the “τTV” scaling for water is still a task for future research.

As a semi-phenomenological model, SL-TS2 is similar to several other two-state models for water (see, e.g., Tanaka, Shi, and co-workers) [7,17,20]—the main difference being the “basic” equation of state for both high- and low-temperature configurations. In this regard, the number of adjustable parameters for the ambient pressure model (seven thermodynamic parameters (Table 1) and five dynamic parameters (Table 2)) is comparable to other approaches. The scaling analysis proposed here, to the best of our knowledge, has not been discussed previously and could help parameterize both thermodynamic and kinetic modeling of supercooled water at varying pressures—at least within the specified region (0–100 MPa).

It is important to note that while SL-TS2 was originally proposed to describe the glass transition in amorphous polymers and organics, here it is applied to events that are well separated from the “true” glass transition (~130–150 K for water). This is due to the low activation energy of the low-temperature state of water (~20–30 kJ/mol) as compared to polymers (~100–300 kJ/mol). Thus, if we consider the ratio *T_g_/T_X_* (where *T_g_* is the glass transition temperature, and *T_X_* is the point where the free energies of the high- and low-temperature states are equal), it would be very close to 1 for polymers and organics, but only ~0.5–0.6 for water.

Finally, we should emphasize that the Casalini–Roland-type scaling (Equation (15) in the Section 4; see below) is only valid at low pressures, far away from the LLCP. The implicit assumption of this scaling is that the high- and low-density states do not change even as the temperature and pressure are modified. As the system is moved closer to the LLCP, the two states would be expected to move closer and closer together. In terms of the SL-TS2 model, this would imply that the parameters $\Delta r=r_{H}-r_{L}$, $\Delta \varepsilon =\varepsilon_{HH}-\varepsilon_{LL}$, $\Delta S_{H}=S_{H,H}-S_{H,L}$, and $\Delta E=E_{H}-E_{L}$ must all approach zero in the vicinity of the LLCP. Elucidating the dependence of those parameters on the pressure and temperature, and interpolating between the near-critical region and the Casalini-Roland scaling region, will be the subject of future research.

Overall, the proposed simple model successfully describes both the equation of state and the transport properties of liquid water in a wide temperature (~180–360 K) and pressure (~0 to 100 MPa) range. The framework, in principle, is flexible enough to allow the model to be extended to higher pressures closer to the LLCP. Another, more challenging problem is extending the proposed approach to integrate the crystalline phase (“ice”), at least in its most common form. These will be topics for future research. 

## 4. Materials and Methods

### 4.1. The Model Density and Specific Volume

**Figure 7:**
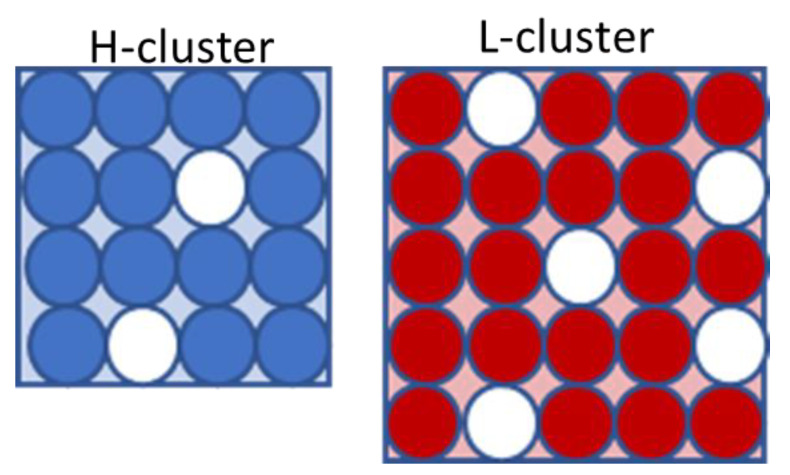
The schematic representation of the L and H clusters within the SL-TS2 model. See text for more details.

We adopted the two-state SL-TS2 formalism that combines the description of the density and relaxation time of an amorphous liquid or glassy material (see Ginzburg et al. [45,47,48]). Within this model, water is considered as an ensemble comprised of two types of “clusters” (or cooperatively rearranging regions (CRRs)) (see Figure 7). The lower-temperature state, L (right panel), is characterized by lower density, i.e., higher volume, while the higher-temperature state, H (left panel), has higher density and lower volume (note that the word “state” here refers not to the macroscopic thermodynamic phase, but to the microscopic local structure). The Gibbs free energy of a system of *N* clusters, including *X* being in the L-state and *(N-X)* being in the H-state, is written as follows:

(5) $$\begin{array}{c} G=k_{B}T\left[X\left(\frac{S_{H,L}}{k_{B}}\right)+(N-X)\left(\frac{S_{H,H}}{k_{B}}\right)\right]\\ \;\;\;\;\;\;\;\;\;\;\;+k_{B}T\left[X\ln\phi_{L}+(N-X)\ln\phi_{H}+Y\ln\phi_{V}\right]\\ \;\;\;\;\;\;\;\;\;\;\;\;\;-\frac{z}{2}\frac{V}{v_{0}}\left[\varepsilon_{HH}\phi_{H}^{2}+2\varepsilon_{HL}\phi_{H}\phi_{L}+\varepsilon_{LL}\phi_{L}^{2}\right]+PV \end{array}$$

Here, *φ_L_* is the volume fraction of the L-clusters, *φ_H_* is the volume fraction of the H-clusters, and *φ_V_* is the volume fraction of the voids or empty spaces (recall that we utilized the Sanchez–Lacombe [63,64,65,66,67] lattice framework); *V* is the total volume, *Y = Vφ_V_* is the total number of voids, *v_0_* is the volume of one lattice site, *z* is the coordination number, and *ε_ij_* (i, j = L, H) represents the van der Waals energies between the neighboring sites. The “internal” entropy of an L-cluster is labeled *S_H,L_*, and that of an H-cluster is labeled *S_H,H_*; the first index “H” refers to the origin of this entropy term as arising from the combinatorics of hydrogen bonds within the cluster (see the Section 3 for more details). Finally, T is the temperature, P is the pressure, and *k_B_* is the Boltzmann constant.

It is convenient to rewrite the free energy in a non-dimensional way, as follows:

(6) $$\begin{array}{l} \tilde{G}\equiv \frac{G}{N\varepsilon *}=\\ \;\;\;\;\tilde{T}\left[\psi \ln\phi_{L}+\left(1-\psi \right)\ln\phi_{H}+\langle r\rangle \left\{\frac{1}{\nu }-1\right\}\ln\phi_{V}\right]\\ \;\;\;\;-\tilde{T}\left[\left(\Delta S_{H}\right)\psi \right]-\frac{\langle r\rangle }{\nu }\left[\phi_{L}^{2}+2\alpha_{LH}\phi_{L}\phi_{H}+\alpha_{HH}\phi_{H}^{2}\right]\\ \;\;\;\;+\frac{\tilde{P}\langle r\rangle }{\nu }+const. \end{array}$$

where we can define $\varepsilon^{*}\equiv z\varepsilon_{LL}$, $\tilde{T}\equiv k_{B}T/\varepsilon^{*}$, $\tilde{P}\equiv Pv_{0}/\varepsilon^{*}$, and introduce two new variables: ψ=X/N, the molar fraction of the L-clusters, and $\nu =1-(Y/V)=1-\phi_{V}$, the “occupancy” (i.e., fraction of non-void sites). Moreover, $\langle r\rangle =r_{L}\psi +r_{H}\left(1-\psi \right)$, $\phi_{L}=\nu \frac{\psi r_{L}}{\langle r\rangle }$, $\phi_{H}=\nu \frac{\left(1-\psi \right)r_{H}}{\langle r\rangle }$, $\phi_{V}=1-\nu$, $\alpha_{LH}=\frac{\varepsilon_{LH}}{\varepsilon_{HH}}$, and $\alpha_{HH}=\frac{\varepsilon_{HH}}{\varepsilon_{LL}}$ (note that $\phi_{V}$ can be thought of as the “free volume”). The volume of the L-cluster is assumed to be equal to *r_L_* lattice units, while that of the H-cluster is assumed to be equal to *r_H_* lattice units. Notably, for water, the higher-temperature state is denser than the lower-temperature state; thus, *r_L_* > *r_H_* (unlike most other materials). Finally, $\Delta S_{H}=S_{H,H}-S_{H,L}$ is the entropy difference between the high- and low-temperature states.

Minimizing the free energy with respect to *ψ* and *ν* leads to the following equations:

(7) $$\ln\frac{\psi }{1-\psi }+\Delta \tilde{S}-\frac{\Delta \tilde{U}+\tilde{P}\Delta \tilde{V}}{\tilde{T}}=0$$

(8) $$\tilde{T}\left[\ln\left(1-\tilde{\rho }\right)+\tilde{\rho }\left\{1-\frac{1}{\langle r\rangle }\right\}\right]+{\tilde{\rho }}^{2}J+\tilde{P}=0$$

where

(9) $$\Delta \tilde{S}=\Delta S_{H}+\ln\frac{r_{L}}{r_{H}}+\frac{r_{H}-r_{L}}{\langle r\rangle }+\left(r_{H}-r_{L}\right)\left(\frac{1}{\nu }-1\right)\ln\frac{1}{1-\nu }$$

(10) $$\begin{array}{l} -\Delta \tilde{U}=\frac{\nu }{{\langle r\rangle }^{2}}\left\{\left(r_{H}-r_{L}\right)\psi^{2}r_{L}^{2}-2\psi r_{L}^{2}r_{H}\right\}\\ \;\;\;\;\;\;\;\;\;\;+2\alpha_{LH}\frac{\nu }{{\langle r\rangle }^{2}}\left\{\left(r_{H}-r_{L}\right)\psi \left(1-\psi \right)r_{L}r_{H}-r_{L}r_{H}\left[\left(1-\psi \right)r_{H}-\psi r_{L}\right]\right\}\\ \;\;\;\;\;\;\;\;\;\;+\alpha_{HH}\frac{\nu }{{\langle r\rangle }^{2}}\left\{\left(r_{H}-r_{L}\right){\left(1-\psi \right)}^{2}r_{H}^{2}+2\left(1-\psi \right)r_{H}^{2}r_{L}\right\} \end{array}$$

(11) $$\Delta \tilde{V}=\frac{\left(r_{H}-r_{L}\right)}{\nu }$$

(12) $$J={\left(\frac{\psi r_{L}}{\langle r\rangle }\right)}^{2}+2\alpha_{LH}\left(\frac{\psi r_{L}}{\langle r\rangle }\right)\left(\frac{\left\{1-\psi \right\}r_{H}}{\langle r\rangle }\right)+\alpha_{HH}{\left(\frac{\left\{1-\psi \right\}r_{H}}{\langle r\rangle }\right)}^{2}$$

Equations (7) through (12) are similar to Equations (6) and (7) in Ref. [45] (the index “L” here corresponds to the index “S” there, and the index “H” here corresponds to the index “L” there), except for two major differences: First, the high-temperature state of water has lower volume than the low-temperature state, i.e., *r_H_ < r_L_*; this means that the parameter $\Delta \tilde{V}$ is negative, and the increase in pressure promotes the high-temperature (not low-temperature) state. Second, because of the above, the translational entropy of the high-temperature (H) state is lower than that of the low-temperature (L) state. This is offset by the hydrogen-bonding combinatorial term $\Delta S_{H}$ > 0. The number of possible arrangements of various hydrogen bonds within the H-cluster is assumed to be significantly greater than that in the L-cluster. 

Given that the glass transition temperature of water is fairly low (*T_g_*~130–160 K) [2,38,40,68], the state of the matter is equilibrium for all temperatures and pressures considered here. The specific volume (i.e., the inverse of the density) is given by $v_{sp}=v_{sp0}\left(P\right)\frac{\langle r\rangle }{r_{L}\nu }$, where $v_{sp0}\left(P\right)$ is the (pressure-dependent) specific volume at zero temperature. Thus, we can determine the density or specific volume unambiguously for any *T* and *P* after solving Equations (7) and (8) for *y* and *ν*. 

### 4.2. The Model’s Relaxation Time and Diffusion

Within the TS2 framework (similar to the Shi-Tanaka-Russo model [20]), the *α*-relaxation time is given by

(13) $$\tau_{\alpha }\left(T,P\right)=\tau_{\infty }\exp\left[\frac{E_{H}}{RT}+\frac{E_{L}-E_{H}}{RT}\psi \left(T,P\right)\right]$$

where *E_H_* is the activation energy for the H-state, and *E_L_* is the activation energy for the L-state. Similar to the case of conventional (non-hydrogen-bonding) amorphous materials, *E_L_* > *E_H_*.

The diffusion coefficient can be estimated as follows:

(14) $$D=a_{0}^{2}/\tau_{\alpha }$$

where *a*_0_ is some characteristic length that is assumed to be temperature-independent but pressure-dependent. Strictly speaking, there is a linear temperature dependence of *D* on *T* in addition to the Arrhenius or super-Arrhenius dependence built into the relaxation time, but here we neglect this weaker dependence. Thus, log(*D*) and −log(*τ_α_*) differ only by an additive constant, to be determined if both the diffusion coefficient and relaxation time are known at any given temperature.

### 4.3. The Dynamic “τTV” Scaling

It has been shown that for many amorphous materials, there is a relationship between the relaxation times for various “corresponding states”, described by the following formula:

(15) $$\tau_{\alpha }\left(T,P\right)=f\left(T{\left[v_{sp}\left(T,P\right)\right]}^{\gamma }\right)$$

where *f(x)* is some material-specific monotonic function. This scaling indicates that the relaxation time depends not on *T* and *P* separately, but on some “state variable” Z(*T*,*P*). The exponent *γ* is usually a positive number, ranging from ~0.5 to ~9 [49,50,51,52]; it is supposed to be independent of *P*, at least within some reasonably broad range. For water, however, this scaling obviously fails because of the non-monotonic temperature dependence of the specific volume at constant pressure. One can imagine two different temperatures *T*_1_ and *T*_2_ such that the right-hand sides are the same, but the left-hand sides are drastically different. The question is whether the corresponding states concept applies to water at all.

Recently, it was demonstrated that the Casalini-Roland scaling (Equation (15)) emerges naturally within the SL-TS2 framework (at least within some reasonably broad pressure range) [45,48]. Here, we follow the same idea. First, we assume that all of the temperature- and energy-type parameters (ε^*^, E_H_, E_L_) depend on the pressure, as follows:

(16) $$Y\left(P\right)=Y\left(0\right)\exp\left(-\alpha_{T}P\right)$$

where *Y* == (ε^*^, E_H_, E_L_), and *α_Τ_* =1/*P*_0,*T*_ [48]. Note that Equation (16) implies that the energy-type parameters decrease upon increasing pressure; this is counterintuitive, but it seems to be the only way to describe the pressure dependence of the density-temperature curves.

Next, we consider the density scale and assume that

(17) $$v_{sp0}\left(P\right)=v_{sp0}\left(0\right)\exp\left(-\alpha_{\rho }P\right)$$

where *α_r_* =1/*P*_0,v_ is the isothermal compressibility. Finally, we further hypothesize that

(18) $$a_{0}^{2}\left(P\right)=a_{0}^{2}\left(0\right)\exp\left(-\alpha_{D}P\right)$$

again, with some positive *α_D_*. Here, and below, we set *a*_0_(0) = 0.14 nm = 1.4 Å.

The “corresponding states” hypothesis can be expressed as follows:

(19) $$\frac{v_{sp}\left(T,P\right)}{v_{sp0}\left(P\right)}=F\left(\frac{T}{T_{X}\left(P\right)}\right)$$

(20) $$D\left(T,P\right)=\frac{a_{0}^{2}\left(P\right)}{\tau_{\alpha }\left(T,P\right)}=a_{0}^{2}\left(P\right)H\left(\frac{T}{T_{X}\left(P\right)}\right)$$

where *T_X_* is defined as the temperature for which *ψ* = 0.5 (equal numbers of H- and L-states). The pressure dependence of *T_X_* is given by Equation (16), such that *T_X_* decreases as *P* is increased. The state variables *y* and *ν* then depend on the combined variable *T/T_X_(P)*—not on *T* and *P* independently.

### 4.4. Experimental Data

To test our models, we used the published experimental data from multiple sources; these data were collected using standard techniques and instruments and were found to reproduce one another fairly well when the measurements were done under similar environmental conditions (i.e., similar pressures and temperatures). The self-diffusion data were obtained from a series of different experiments from the literature. In detail, at atmospheric pressure, we considered data from Yoshida et al. [69] in the 303–450 K temperature range, from Simpson and Carr [70] for 273 K < *T* < 373 K, from Price et al. [71] for 238 K < *T* < 298 K, and from Xu et al. [72] for 180 K < *T* < 262 K. The first three works consider the nuclear magnetic resonance (NMR) pulsed-field-gradient spin-echo method, allowing the evaluation of the self-diffusion coefficient from the trend of the spin-echo decay as a function of the intensity (or duration) of the perturbing magnetic gradient pulse [73]. In contrast, Xu et al. [72] used a pulsed laser for unfreeze ice and perform the measurement on liquid water by studying the growth rate of crystalline ice and applying the well-known Wilson-Frenkel model [74]. At higher pressure, we considered the self-diffusion data published by Prielmeier et al. [75], including data in the higher-temperature regime (*T* > 273 K) measured by Weingärtner [76] and by Tyrrell and Harris [77]. All of them used the NMR pulsed-field-gradient spin-echo method.

The density data were also obtained from different experiments; in particular, we considered atmospheric-pressure data from Grindley et al. [78] for 298 K < *T* < 423 K, from Kell and Whalley [79] for 273 K < *T* < 373 K, from Hare and Sorensen [80] in the 239–313 K temperature range, from Mallamace et al. [81] for 183 K < *T* < 260 K, and from Erko et al. [82] for 170 K < *T* < 220 K (the latter two were measured in a confining environment to prevent the water from freezing). In detail, Grindley et al. measured the water’s specific volume by means of a piezometer [78]; the data of Kell and Whalley were derived from the sound speed data of Wilson et al. [83]; Hare and Sorensen [80] measured the water density in glass capillaries with a 25-micron internal diameter; the data of Mallamace et al. [81] were obtained from Fourier-transform infrared measurements on water confined in silica nanotubes with a diameter of 14 angstrom, and those of Erko et al. [82] were obtained by combining small-angle scattering of X-rays and neutrons on water confined within cylindrical pores of ordered nanoporous material with a pore size of 34 Å. At higher pressures, we considered the density data of Mishima (who measured the specific volume of water droplets 1–10 mm in size [84]) at the lowest temperatures, and those of Kell and Whalley [79] and Grindley et al. [78] at the highest temperatures. We considered also few data above 423 K coming from the IAPWS-95 formulation [85], and we performed a polynomial interpolation for the temperature ranges with superimposed values.

## 5. Conclusions

We applied the “two-state, two-(time)scale” Sanchez–Lacombe model (SL-TS2) to describe the equation of state and diffusion coefficient of both equilibrium and supercooled liquid water. The model showed a good agreement with the experimental data for the ambient-pressure measurements in a broad temperature range (180–360 K). We also discovered non-trivial scaling rules similar to the Casalini-Roland “τTV” scaling in “conventional” glass-formers; using this scaling, we successfully described the density and diffusion coefficient at multiple pressures (0–100 MPa). The results could provide a new description of both the equilibrium and transport properties of liquid water and, thus, may be of interest in many applications in the chemical and other industries.

## Figures and Tables

**Table 1:** The density and diffusion coefficient scaling parameters. See text for more details.

Parameter	Units	Value
a_r_	(MPa)^−1^	5.48 × 10^−4^
a_T_	(MPa)^−1^	9.35 × 10^−4^
a_D_	(MPa)^−1^	7.78 × 10^−3^

**Table 2:** Two-state Sanchez–Lacombe parameters for amorphous water.

Parameter	Units	Value
T*	K	734.1
*r_L_*		56.5
*r_H_*		50.6
*α_HH_*		1.0
*α* *_HΛ_*		1.0
Δ*S_H_*		18.5
*V_sp,0_*	cm^3^/g	1.073

**Table 3:** Parameters for TS2 and modified TS2 (“mTS2”). See text for more details.

Parameter	Units	Value
_τ∞_	s	4.0 × 10^−13^
*E_H_*	kJ/mol	6.7
**TS2**		
*E_L_*	kJ/mol	20.1
**mTS2**		
*E_L,min_*	kJ/mol	19.5
*E_L,max_*	kJ/mol	28.1
*k*		25.0

## Data Availability

The data and models (Excel files) can be requested from the authors.
